# Polynomial Regression on Lie Groups and Application to *SE*(3)

**DOI:** 10.3390/e26100825

**Published:** 2024-09-27

**Authors:** Johan Aubray, Florence Nicol

**Affiliations:** Ecole Nationale de l’Aviation Civile, Université de Toulouse, 7, Avenue Edouard Belin, 31400 Toulouse, France; florence.nicol@enac.fr

**Keywords:** Lie groups, *SE*(*n*), regression, Riemannian manifolds, affine connection, linear connection, air traffic management

## Abstract

In this paper, we address the problem of estimating the position of a mobile such as a drone from noisy position measurements using the framework of Lie groups. To model the motion of a rigid body, the relevant Lie group happens to be the Special Euclidean group SE(n), with n=2 or 3. Our work was carried out using a previously used parametric framework which derived equations for geodesic regression and polynomial regression on Riemannian manifolds. Based on this approach, our goal was to implement this technique in the Lie group SE(3) context. Given a set of noisy points in SE(3) representing measurements on the trajectory of a mobile, one wants to find the geodesic that best fits those points in a Riemannian least squares sense. Finally, applications to simulated data are proposed to illustrate this work. The limitations of such a method and future perspectives are discussed.

## 1. Introduction

Estimating the position of an aircraft in the context of air traffic management (ATM) is necessary in two situations. In the first one, the goal is to present air traffic controllers with a clean image of the aircraft positions in their sectors. In this case, only the past trajectory is known, and one wants to give the best estimate of the current position. This is the radar tracking problem that has been dealt with for decades using Kalman filtering and its extensions [1], which has recently been studied in the framework of Lie groups [2,3]. In [4,5], the symmetries of the state space seen as a manifold are used to improve estimation. In the second situation, a database of full or partial trajectories is available so that the estimate of a position may be computed using past and future measurements. The corresponding noise removal procedure is known as regression and generally relies on a simple, often local model of the trajectory whose parameters are estimated so as to minimize a least squares criterion.

When the data belong to a Euclidean space, statistical regression analyses are usually of two kinds: parametric methods such as linear and polynomial regression and non-parametric methods such as kernel-based technique, spline smoothing, and local polynomial regression. As an alternative, due to the functional nature of trajectories mapping time to position, the problem arising from the targeted application may also be described in the general framework of functional data statistics in which mobile trajectories are functional objects belonging to a given Hilbert space. Usually, in the absence of the notion of density probability in the original infinite-dimensional Hilbert space, the classical approach is to project on a finite-dimensional subspace. However, this works only if it is possible to find a suitable basis in low dimension representing well all the possible trajectories, which is not the case in the context of highly maneuvering aircraft.

Rather than considering trajectories in the state space, we may use the framework of Lie groups by studying the motion of the mobiles in another representation space. Lie groups are continuous groups of transformations such as scalings, rotations, and translations. For poses of a rigid object, the appropriate Lie group is the Special Euclidean group SE(n), which has been used in the last few years in the fields of navigation [6,7,8]; robotics [9,10]; computational anatomy [11,12,13,14]; automation and control theory [15,16,17], which explains concepts involved in calculus on manifolds such as covariant derivatives and curvature; and signal processing [18,19,20], among others.

Recently, non-parametric estimation methods on Lie groups have also been explored, such as [21], in which the authors developed non-parametric kernel-based regression where the response variable is real-valued, and the Lie group-valued predictors are contaminated by measurement errors.

Parametric models have also been studied: the analogies of Riemannian *k*th-order polynomials with the theory of geodesics in Riemannian manifolds were studied in [22] and further generalized regarding splines on manifolds in [23]. Riemannian polynomials [24] as well as manifold-valued spline schemes based on Bézier curves [25,26] have also been investigated. Our work is guided by the applications and is situated in a parametric framework of the geodesic regression method developed in [27]. This method was extended in [24] to higher-order polynomials on Riemannian manifolds, which include Lie groups with a left or right invariant Riemannian metric. The algorithm for geodesic and polynomial regression is explicitly derived in SO(3). Our goal is to follow the same idea by modeling the motion of rigid bodies and rigid transformations with the Special Euclidean group SE(n), *n* = 2 or 3. This group, unfortunately, does not provide a bi-invariant metric (a metric may be left- or right-invariant but not both at the same time) and, thus, does not allow for regression consistent with group operations, meaning that regression on a given sample may be similar to the regression applied to the same sample either translated on the left or on the right.

In [24], geodesic and polynomial calculations were carried out using the Levi–Civita connection, the main advantage of which is that it is compatible with the metric, and the calculations are indeed performed using that connection only. In the present contribution, instead of introducing Lagrange multipliers, a more straightforward approach is taken, by reasoning in terms of differential forms (minimizing an objective function means finding the points that cancel its first differential). Then, we extend this principle to an arbitrary connection since many applications use connections other than the Levi–Civita connection. For example, Ref. [28] uses Cartan–Schouten connections to express geodesics in Lie groups as one-parameter subgroups. Similarly, α-connections [29] are often used in statistical models on manifolds. The advantage of using any given connection can be seen in two ways: either it simplifies the writing of the problem, or it allows us to express a non-intrinsic quantity, i.e., one whose writing depends on a specific coordinate system (e.g., this is the case for polynomial curves, which are expressed differently depending on the coordinate system chosen).

The paper is organized as follows: In the second section, the elements of differential geometry necessary to lay the theoretical background are discussed. The third section presents the notion of a polynomial function in a Lie group using jet bundles. The fourth section provides a mathematical formulation for the regression problem using differential forms. Finally, several applications to simulated data are proposed. The limitations of the method are discussed, and future perspectives to improve the estimation are proposed.

## 2. Elements of Differential Geometry

### 2.1. Manifolds, Lie Groups, and Vector Bundles

We assume the reader is familiar with the definition of a manifold as a submanifold of $R^{n}$ and recall the definition of an abstract manifold here:

**Definition** **1.** *An n-dimensional smooth manifold is a second countable, Hausdorff set M endowed with an equivalence class of n-dimensional atlases of class $C^{\infty }$ on M. An n-dimensional atlas of class $C^{\infty }$ on M is a set of pairs ${\left\{\left(U_{i},\phi_{i}\right)\right\}}_{i\in I}$ satisfying the following axioms:*

- *Each $U_{i}$ is an open subset of M and, $M\subset {⋃}_{i}U_{i}$;*
- *Each $\phi_{i}$ is a bijection from $U_{i}$ into an open subset $\phi_{i}\left(U_{i}\right)$ of $R^{n}$, and $\phi_{i}\left(U_{i}⋃U_{j}\right)\subset R^{n}$ is open for every i and j;*
- *For every pair (i,j), the map $\phi_{j}\circ \phi_{i}^{-1}:\phi_{i}\left(U_{i}\cap U_{j}\right)\to \phi_{j}\left(U_{i}\cap U_{j}\right)$ is a $C^{\infty }$ diffeomorphism.*

A Lie group inherits the algebraic properties of a group and the topological properties of a manifold.

**Definition** **2.** *A Lie group G is a smooth manifold and a group such that the multiplication m:G×G→G,(x,y)↦x∘y and the inversion $i:G\to G,x\mapsto x^{-1}$ are smooth operations.*

We shall use the following notations:

- $L_{g}:G\to G,x\mapsto gx$ for the left translation;
- $R_{g}:G\to G,x\mapsto xg$ for the right translation;
- e∈G for the unit element.

**Definition** **3.** *Let E,M be smooth manifolds. A real vector bundle on M is a triple $\left(E,\pi ,M\right)$ with π:E→M a smooth onto mapping such that*

(*i*) *For all p∈M, $\pi^{-1}\left(p\right)$ is a real vector space isomorphic to $R^{k}$, k a fixed integer;*
(*ii*) *For all p∈M, there exists an open set U⊂M,p∈U and a diffeomorphism $\phi_{U}:U\times R^{k}\to \pi^{-1}\left(U\right)$ such that*
  -
$\forall q\in U,v\in R^{k},\;\pi \left(\phi_{U}\left(q,v\right)\right)=q;$

  - *∀q∈U, the mapping $v\in R^{k}\mapsto \phi_{U}\left(q,v\right)$ is an isomorphism on $\phi^{-1}\left(q\right).$*

**Remark** **1.** *Definition 3 can be utilized verbatim to define vector bundles with other fields of scalars, particularly complex or quaternionic vector bundles. However, this additional generality will not be required for the purposes of this paper.*

**Notation** **1.** A bundle (E,π,M) is often represented by its total space *E* along, π being implicit.

**Definition** **4.** *Let U⊂M be an open set and (E,π,M) a vector bundle on M. A smooth mapping s:U→E such that $\forall p\in M,\;\pi \left(s(p)\right)=p$ is called a local section. When U=M, s is designated as a global section or simply a section.*

**Proposition** **1.** *Let U⊂M be an open set and (E,π,M) a vector bundle on M. The set of sections on U has the structure of a real vector space and a $C^{\infty }\left(U,R\right)$ module.*

**Notation** **2.** The $C^{\infty }\left(U,R\right)$-module of local sections on *U* is designated by $\Gamma \left(U;E\right).$ When U=M, it is simply denoted by Γ(E).

**Remark** **2.** *One can define equivalently a vector bundle as the sheaf generated by the local sections.*

**Remark** **3.** *A vector bundle of dimension 1 is called a line bundle. Its sections are the $C^{\infty }\left(M,R\right)$-mappings.*

**Definition** **5.** *A triple (M,E,∇) with M a smooth manifold, E a vector bundle on M and ∇ a Koszul connection on E is called a gauge structure.*

**Proposition** **2.** *Let E,F be two vector bundles over M. The pointwise operations on sections ⊕,⊗,hom(·,·) define the respective vector bundles E⊕F,E⊗F,hom(E,F).*

**Proof.**  See [30], Theorem 6.2, p. 67.    □

**Definition** **6.** The hom bundle with F=R is called the dual bundle of E and is denoted by $E^{*}.$

### 2.2. Koszul Connections

**Definition** **7.** *Let (E,π,M) be a vector bundle over M. A Koszul connection is an R-linear mapping:*

$$\nabla :\Gamma \left(E\right)\to \Gamma \left(TM^{*}⊗E\right)$$

*such that*

(1) $$\forall s\in \Gamma \left(E\right),\forall f\in C^{\infty }\left(M,R\right),\;\nabla \left(fs\right)=df⊗s+f\nabla s$$

*A Koszul connection is often called a covariant derivative.*

**Remark** **4.** *Applying Definition 7 on a local frame $\left\{e_{i},i=1\ldots m\right\}$ of E yields the so-called Christoffel symbols:*

$$\forall k,j\in 1,\ldots ,m,i=1\ldots \dim\left(M\right),\nabla_{\partial_{i}}e_{j}=\Gamma_{ij}^{k}e_{k}$$

*with $\partial_{i},i=1\ldots \dim\left(M\right)$ a basis of TM. The Christoffel symbols obviously uniquely characterize the connection ∇.*

**Remark** **5.** *There is no reason for a locally constant section to vanish under the action of a Koszul connection.*

**Definition** **8.** *Given a local frame of sections $\left\{e_{i}\right\}$, the connection forms are the one forms $\omega_{j}^{i}$ such that*

(2) $$\nabla e_{j}=e_{i}⊗\omega_{j}^{i}$$

**Proposition** **3.** *If E,F are two vector bundles and $\nabla_{E},\nabla_{F}$ are, respectively, connections on E,F, there exist two canonical connections:*

(3) $$\begin{array}{cc} \; & \nabla_{E}⊕\nabla_{F}:s_{E}⊕s_{F}\mapsto \left(\nabla_{E}s_{E}\right)⊕\left(\nabla_{F}s_{F}\right), \end{array}$$

(4) $$\begin{array}{cc} \; & \;\nabla_{E}⊗\nabla_{F}:s_{E}⊗s_{F}\mapsto \left(\nabla_{E}s_{E}\right)⊗s_{F}+s_{E}⊗\left(\nabla_{F}s_{F}\right). \end{array}$$

**Proposition** **4.** *Let E be a vector bundle over M and let $E^{★}$ be its dual bundle. If ∇ is a Koszul connection on E, then the dual-connection $\nabla^{★}$ on $E^{★}$ is defined as follows:*

(5) $$\nabla_{X}^{★}\left(\omega \right)\left(Y\right)=X\left(\omega (Y)\right)-\omega \left(\nabla_{X}Y\right),\;X,Y\in \Gamma \left(TM\right),\omega \in \Gamma \left(E^{★}\right).$$

**Definition** **9** ([31]). *Let $f\in C^{\infty }\left(M,R\right)$, that is f is a section of the trivial bundle M×R. The covariant derivative of f, still denoted by ∇f, is defined as:*

(6) ∇f=df.

Propositions 3 and 4 and Definition 9 can be combined in order to obtain the covariant derivative of arbitrary tensors.

**Definition** **10.** *Let $\left(E,\pi_{E},M\right),\left(F,\pi_{F},N\right)$ be two vector bundles. A bundle morphism is a couple of mappings $\tilde{\phi }:E\to F,\phi :M\to N$ such that, for all x∈M, the mapping $u\in \pi_{E}^{-1}\left(x\right)\to \phi_{F}^{-1}\left(\phi \left(x\right)\right)$ is linear, which makes the next diagram commute:*

(7) $$\begin{array}{ccccc} E & & \overset{\tilde{\phi }}{\to } & F & \\ ↓ & \pi_{E} & & ↓ & \pi_{F}\\ M & & \overset{\phi }{\to } & N & \end{array}$$

**Definition** **11.** Let ϕ:M→N be a smooth mapping and $\left(F,\pi_{F},N\right)$ a vector bundle on N. The pullback bundle $\phi^{*}F$ is the vector bundle on M generated by the local sections of the form x↦s(ϕ(x)) where s is a local section of F. Furthermore, there exists a bundle morphism $\tilde{\phi }:E\to F$ sending a generating section s∘ϕ to s.

**Definition** **12.** *Let M,N be smooth manifolds and ϕ:M→N a smooth mapping. Let E be a vector bundle on N equipped with a Koszul connection ∇. The pullback connection of ∇ by ϕ, designated by $\nabla^{\phi }$, is defined by generating sections s∘ϕ as follows:*

(8) $$\nabla_{X}^{\phi }s\circ \phi =\nabla_{D\phi X}s$$

*where X∈TM.*

**Remark** **6.** *The quantities in Equation (8) depend only on $X\left(p\right)\in T_{p}M$ and $T_{\phi (p)}N.$*

### 2.3. Covariant Derivatives and Parallel Sections

**Definition** **13.** *Let M be a smooth manifold, E a vector bundle on M and ∇ a Koszul connection on E. Let γ:]0,1[→M a smooth mapping, that is a curve on M. The covariant derivative of s∈Γ(E) along γ is the mapping:*

(9) $$t\in ]0,1[\to \nabla_{1}^{\gamma }s\circ \gamma \left(t\right)$$

*where* 1 *is the unit constant vector in ]0,1[.*

**Notation** **3.** The covariant derivative of a section *s* along a curve γ is often denoted as follows:

$$\nabla_{\dot{\gamma }}s$$

**Proposition** **5.** *With the same writing conventions as in Definition 13, the covariant derivative of a section $s=s^{i}e_{i}$ expressed in a local frame $\left(e_{1},\ldots ,e_{n}\right)$ is given as follows:*

(10) $$ds^{i}\left(\dot{\gamma }\left(t\right)\right)e_{i}+\Gamma_{ij}^{k}s^{i}{\dot{\gamma }}^{j}\left(t\right)e_{n}$$

**Proof.**  This result is a direct application of Definition 12 with ϕ=γ.    □

**Remark** **7.** *The first term in Equation (10) can be written by a common abuse of notation:*

(11) $$\frac{d}{dt}s^{i}\left(t\right)e_{i}$$

*where $s^{i}e_{i}$ stands for the section in the pullback bundle associated to s.*

**Definition** **14.** *Let (M,E,∇) be a gauge structure and let γ:]0,1[→M be a smooth curve. A section s∈Γ(E) is said to be ∇-parallel along γ if*

$$\nabla_{\dot{\gamma }}s=0.$$

**Remark** **8.** *A parallel section is, in a local frame, the solution of an ordinary differential equation (ODE):*

(12) $$\frac{d}{dt}s^{k}\left(t\right)+\Gamma_{ij}^{k}s^{i}{\dot{\gamma }}^{j}\left(t\right)=0,\;k=1\ldots n$$

*This gives a practical way to determine a parallel section from an initial condition.*

**Notation** **4.** The parallel transport of a section *s* from *a* to *b* will be denoted $\prod_{a}^{b}s$.

Given a gauge structure (M,TM,∇), x∈M and a tangent vector $X\in T_{x}M$, the next ODE locally defines a smooth curve γ:

(13) $$\left\{\begin{array}{cc} \; & \dot{\gamma }\left(t\right)=s\circ \gamma \left(t\right)\\ \; & \nabla_{\dot{\gamma }}s=0\\ \; & \gamma (0)=x \end{array}\right.$$

**Definition** **15.** *Let (M,TM,∇) be a gauge structure. For any x∈M, there exists an open neighborhood U of 0 in $T_{x}M$ such that the curve γ defined by Equation (13) exists to time *1* for a tangent vector X∈U. The mapping is as follows:*

(14) $$X\mapsto \exp_{x}X=\gamma \left(1\right)$$

*where γ is the solution curve to Equation (13) is called the exponential map.*

**Definition** **16.** *Let $\left(e_{1},\ldots ,e_{n}\right)$ be a basis of $T_{x}M$ such that the exponential map exists for all the $e_{i},\;i=1\ldots n.$ The mapping*

(15) $$\left(t_{1},\ldots ,t_{n}\right)\in {\left[0,1\right]}^{n}\mapsto \exp_{x}\left(\sum_{i=1}^{n}t_{i}e_{i}\right)$$

*defines a local coordinate system in a neighborhood of x called the normal coordinates at x.*

**Remark** **9.** *By the very definition of a flow, the following is deduced:*

(16) $$\frac{d}{dt}\exp_{x}tX=s\left(\gamma (t)\right)$$

*with γ,s is the solution to ODE (13). Taking the second derivative is slightly awkward since the corresponding tangent vector would lie in TTM. However, it makes sense to consider the covariant derivative along γ:*

(17) $$\nabla_{\dot{\gamma }}s=0.$$

*The normal coordinate system thus has a vanishing second (covariant) derivative. The generalization of this property to higher covariant derivatives will be used later as an extension of the notion of polynomial curves.*

**Notation** **5.** A normal coordinate system at *x* is often denoted by $x^{i},\;i=1\ldots n$ and the associated vector fields by $\partial_{i}.$ A vector field *X* is said to be a coordinate vector field if it can be written as $X=a^{i}\partial_{i}$ with $a_{i},i=1\ldots n$ constant mappings [31].

**Proposition** **6.** *Any two coordinate vector fields X,Y have a vanishing commutator [X,Y]=0.*

**Proof.**  Writing $X=X^{i}\partial_{i},Y=Y^{i}\partial_{i}$, and using the fact that $X^{i},Y^{i},i=1\ldots n$ are constant mappings, the following is derived:

$$\left[X,Y\right]=\left(\left(\partial_{j}Y^{i}\right)X^{j}-\left(\partial_{j}X^{i}\right)Y^{j}\right)\partial_{i}=0$$

   □

### 2.4. Musical Isomorphisms

In the sequel, we will often have to toggle between matching differential forms and vectors. We define the musical isomorphisms using the metric *g*.

**Definition** **17.** Let (M,g) be a Riemannian manifold. Let $\alpha \in T^{*}M$. $\alpha^{♯}$ is the only element of TM such that, for all X∈TM,

(18) $$\alpha \left(X\right)=g\left(\alpha^{♯},X\right)$$

*and we note it as $\alpha^{♯}:=♯\left(\alpha \right)$.*

In a similar way, for Y∈TM, $Y^{♭}$ is the only element of $T^{*}M$ such that, for all X∈TM,

(19) $$Y^{♭}\left(X\right)=g\left(X,Y\right)$$

*and we note it as $Y^{♭}:=♭\left(Y\right)$.*

Both isomorphisms can be expressed in local coordinates:

$$\begin{array}{cc} \alpha^{♯} & =♯\left(\alpha \right)=♯\left(\alpha_{i}e^{i}\right)=\underset{X^{i}}{\underset{︸}{g^{ij}\alpha_{j}}}e_{i},\\ X^{♭} & =♭\left(X\right)=♭\left(X^{i}e_{i}\right)=\underset{\alpha_{i}}{\underset{︸}{g_{ij}X^{j}}}e^{i}. \end{array}$$

where $g_{ij}$ and $g^{ij}$ are, respectively, the i,j coefficient of the metric matrix and the i,j coefficient of the inverse of the metric matrix.

## 3. Manifold-Valued Polynomial Functions

Here, we provide the basic elements on how a manifold-valued polynomial function P:I⊂R→M can be well defined.

As a consequence of the Taylor formula, the notion of a polynomial function of degree *k* on $R^{n}$ is defined as a smooth mapping $P:R^{n}\to R$ such that

$$\frac{\partial^{\left|I\right|}P}{\partial^{I_{1}}x_{1}\ldots \partial^{I_{n}}x_{n}}=0$$

for any multi-index $I=(I_{1},\ldots ,I_{n})$ such that $\left|I\right|=\sum_{i=1}^{n}I_{i}>k$.

While it is meaningful to speak about higher derivatives only for vector space-valued maps, things are more complicated for manifold-valued maps. This kind of property may hold locally, that is, for example, if a function has a finite length Taylor expansion in terms of a local coordinate system, but generally, it fails globally, due to the transition functions between local charts. Two possibilities exist to overcome this:

- Ref. [32] restricts the Hessian to the kernel of $df_{p}$ so that the second derivative makes sense through the transition maps of the manifold;
- A possibility to bypass this [33] is to consider directly a *k*-th order Taylor series through jet bundles: let $\gamma_{1},\gamma_{2}$ be two curves I⊂R→M such that $\gamma_{1}\left(0\right)=\gamma_{2}\left(0\right)=p$ and (U,ϕ) a local chart such that ∀i∈1…k,
  $${\left.\frac{d^{i}}{dt^{i}}\right|}_{t=0}\phi \circ \gamma_{1}\left(t\right)={\left.\frac{d^{i}}{dt^{i}}\right|}_{t=0}\phi \circ \gamma_{2}\left(t\right)$$
  This relation is an equivalence relation in which equivalence classes are called *k*-jets at *p*, noted $J_{p}^{k}$. The space of all *k*-jets of the bundle (E,π,M) is the union of *k*-jets at *p* over all the points of M.
  The set of all *k*-jets at *p* can be given a differentiable structure in a “tower-like” bundle endowed with adapted coordinates, in which
  $$\forall m\in 1,\ldots ,k,\forall p\in M,\;\pi^{m}:J^{m}\to J^{m-1},\;{\left[\gamma \right]}_{p}^{m}\mapsto {\left[\gamma \right]}_{p}^{m-1}$$
  are projections from one level of the bundle to the one under.

$$\begin{array}{cc} J^{m} & \\ ↓ & \pi^{m}\\ J^{m-1} & \\ ↓ & \pi^{m-1}\\ \ldots & \\ ↓ & \pi^{1}\\ J^{0}=E & \\ ↓ & \pi \\ M & \end{array}$$

This work uses the second approach, though it will not be explicitly reminded in the sequel.

## 4. Optimality Conditions

In this section, the first and second-order local optimality conditions for a real-valued mapping with domain a Riemannian manifold are briefly introduced.

**Lemma** **1.** *Let M be a smooth manifold and f:M→R a smooth mapping. For p∈M to be a local minimum of f, it is necessary that p be a critical point of f, which is the case if and only if the differential of f at p vanishes: $df_{p}=0$.*

**Proof.**  See [34]    □

In fact, if γ:]−ϵ,+ϵ[→M is a smooth curve on *M* such that γ(0)=p, then $df_{p}\left(\dot{\gamma }\left(0\right)\right)$ is the first-order variation of *f* in the direction of the tangent vector $\dot{\gamma }\left(0\right)\in T_{p}M.$ This approach can be extended by iterating the derivative.

**Definition** **18.** *Let M be a smooth manifold: a retraction is a smooth map*

$$R:TM\to M,\left(x,v\right)\mapsto R_{x}\left(v\right)$$

*such that for any curve $c:]-\varepsilon ,\varepsilon [\to M,t\mapsto R_{x}\left(tv\right)$, we have c(0)=x and $c^{\prime }\left(0\right)=v$.*

**Remark** **10.** *The Riemannian exponential $\left(x,v\right)\mapsto \exp_{x}\left(v\right)$ and the linear map (x,v)↦x+v are examples of a retraction.*

From this, a possible algorithm for optimization on manifolds is the Riemannian gradient descent (RGD) explained in Algorithm 1, taken from [34]:
**Algorithm 1:** Riemannian gradient descent
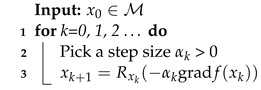


where *R* is a retraction.

## 5. The Geodesic Regression Problem

### 5.1. Polynomial Curves

We recall that in a Lie group *G* with its Lie algebra g, there is a way to easily construct a left-invariant metric from any given inner product on g, which itself can be derived from the Euclidean inner product, since $g≅R^{\dim G}$:

(20) $$⟪{\;X_{g},Y_{g}⟫\;}_{g}=\langle T_{g}L_{g^{-1}}X_{g},T_{g}L_{g^{-1}}Y_{g}\rangle$$

Please note that any symmetric positive-definite matrix may be used to produce different left invariant metrics:

(21) $$⟪{\;X_{g},Y_{g}⟫\;}_{A,g}=\langle T_{g}L_{g^{-1}}AX_{g},T_{g}L_{g^{-1}}AY_{g}\rangle$$

Likewise, a right-invariant metric can also be defined. A minimizing geodesic between two points $g_{0},g_{1}$ with respect to $⟪{\;\cdot ,\cdot ⟫\;}_{g}$ is a curve γ:[0,1]→G, such that $\gamma \left(0\right)=g_{0},$$\gamma \left(1\right)=g_{1}$n minimizing the functional

(22) $$L\left(\gamma \right)=\frac{1}{2}\int_{0}^{1}⟪{\;\dot{\gamma }\left(t\right),\dot{\gamma }\left(t\right)⟫\;}_{g}dt$$

Due to the left invariance of the metric, it is enough to seek a solution to the reduced problem:

(23) $$L\left(X\right)=\frac{1}{2}\int_{0}^{1}⟪{\;\dot{X}\left(t\right),\dot{X}\left(t\right)⟫\;}_{e}dt$$

where X:]−ϵ,ϵ[→g is a g-valued curve. It is well known that *X* satisfies the so-called Euler–Arnold equation [35]:

(24) $$\dot{X}\left(t\right)={\mathrm{ad}}_{X(t)}^{t}X\left(t\right)$$

From the solution of Equation (24), the original geodesic is reconstructed with the help of the left translation

(25) $$\dot{\gamma }\left(t\right)=T_{e}L_{\gamma (t)}X\left(t\right)$$

which simplifies in a matrix Lie group as follows:

(26) $$\gamma \left(t\right)=\exp\left(\int_{0}^{t}X\left(u\right)du\right)$$

Minimizing geodesics are Riemannian equivalents to straight lines and are thus the natural object to consider for extending the linear regression to a Lie group *G*. Furthermore, if ∇ is a connection on *G*, then a minimizing geodesic γ has to satisfy the following:

(27) $$\nabla_{\dot{\gamma }\left(t\right)}\dot{\gamma }\left(t\right)=0$$

thus, the Riemannian equivalent to a degree *k* polynomial is a curve γ such that

(28) $${\left(\nabla_{\dot{\gamma }\left(t\right)}\right)}^{k}\dot{\gamma }\left(t\right)=0$$

Here again, by the left invariance of the metric, the connection in $T_{g}G$ has to be considered only on the basis of left-invariant vector fields $T_{e}L_{g}e_{1},\ldots ,T_{e}L_{g}e_{n}$. The connection restricted to elements of the Lie algebra will be denoted $\bar{\nabla }$ in the sequel.

Applying the Koszul formula to the basis vectors $\left(e_{1},\ldots e_{n}\right)$ of g, the following is obtained:

(29) $$\Gamma_{ij}^{k}=\frac{1}{2}\left(c_{ij}^{k}+c_{ki}^{j}-c_{jk}^{i}\right)$$

where the $c_{ij}^{k}=\langle \left[e_{i},e_{j}\right],e_{k}\rangle$ are the structure constants and the $\Gamma_{ij}^{k},\left(i,j,k\right)\in 1,\ldots ,n$ are the Christoffel symbols of the connection.

Using this form, one can compute a degree *p* polynomial curve *X* in g by solving an augmented differential equation

(30) $$v_{1}\left(t\right)=\nabla_{X(t)}v_{0}\left(t\right),v_{2}\left(t\right)=\nabla_{X(t)}v_{1}\left(t\right),\ldots ,\nabla_{X(t)}v_{p-1}\left(t\right)=0$$

with the convention $v_{0}=X$. Please note that the previous system can be written in the following form:

(31) $$\frac{d}{dt}\left(\begin{array}{c} v_{0}\\ \vdots \\ v_{p-1} \end{array}\right)=F\left(v_{0},\ldots ,v_{p-1}\right)$$

where the vector $v_{0},\ldots v_{p-1}$ is in $R^{pn}$, with *n* the dimension of g as a real vector space.

### 5.2. Regression in a Lie Group

An extended notion of parametric linear regression on Riemannian manifolds, called intrinsic geodesic regression, has been independently developed in [27,36]. Let $\left(y_{1},\ldots ,y_{N}\right)$ be a set of points representing the trajectory of the mobile on a Riemannian manifold (M,g). The idea is to find a geodesic curve γ(t) on the manifold that best fits those points in a "linear" manner at known times $t_{1},\ldots ,t_{N}$. The estimate is found by minimizing a least squares criterion based on a Riemannian distance between the model γ(t) and the data.

More formally, the geodesic regression model is expressed in [27] as follows:

$$Y={\mathrm{Exp}}_{\gamma (t)}\left(\varepsilon \right)={\mathrm{Exp}}_{{\mathrm{Exp}}_{p}\left(tv\right)}\left(\varepsilon \right)$$

where $\gamma \left(t\right)={\mathrm{Exp}}_{p}\left(tv\right)$ is the geodesic curve given by the initial conditions γ(0)=p and $\dot{\gamma }\left(0\right)=v$, and ε denotes a Gaussian random variable taking values in the tangent space at γ(t).

In [24], this geodesic regression model is generalized to a polynomial one

$$Y={\mathrm{Exp}}_{\gamma (t)}\left(\varepsilon \right)$$

where the curve γ(t) is a Riemannian polynomial of order *k*, as defined by Equation (28). The Riemannian least mean squares estimate is obtained by minimizing the next criterion over the decision vectors $\left(\gamma \left(0\right),\dot{\gamma }\left(0\right),\ldots ,{\dot{\gamma }}^{\left(i\right)}\left(0\right),\ldots ,{\dot{\gamma }}^{\left(k\right)}\left(0\right)\right)$ as follows:

(32) $$E\left(\gamma \right)=\frac{1}{N}\sum_{j=1}^{N}d{\left(\gamma \left(t_{j}\right),y_{j}\right)}^{2}$$

where *d* is a geodesic distance between the model $\gamma (t_{j})$ and the data $y_{j}$, j=1,…,N.

This minimization problem is then performed under the constraints that the model curve γ has, in the geodesic regression problem, a parallel first derivative, namely $\nabla_{\dot{\gamma }\left(t\right)}\dot{\gamma }\left(t\right)=0$, or, more generally, a *k*-th order polynomial constraint ${\left(\nabla_{\dot{\gamma }\left(t\right)}\right)}^{k}\dot{\gamma }\left(t\right)=0$ that is uniquely solved by adding initial conditions $\gamma \left(0\right),\dot{\gamma }\left(0\right),\ldots ,{\dot{\gamma }}^{\left(i\right)}\left(0\right),\ldots ,{\dot{\gamma }}^{\left(k\right)}\left(0\right)$.

Using properties of translation given by Equations (23) and (25) in a Lie group, by denoting $X_{1},\ldots ,X_{i},\ldots ,X_{k}$ the *i*-th order velocities of in g, we obtain the “forward” polynomial equations

(33) $$\left\{\begin{array}{cc} \frac{d}{dt}\gamma \left(t\right) & =\gamma \left(t\right)X_{1}\left(t\right)\\ \; & \;\;\vdots \\ \frac{d}{dt}X_{i}\left(t\right) & ={\bar{\nabla }}_{X_{1}}X_{i}\left(t\right)+X_{i+1}\left(t\right)\\ \; & \;\;\vdots \\ \frac{d}{dt}X_{k}\left(t\right) & ={\bar{\nabla }}_{X_{1}}X_{k}\left(t\right) \end{array}\right.$$

Let us now move to minimizing the criterion given by Equation (32). The next lemma is a well-known result that can be proven using normal coordinates.

**Lemma** **2.** *Let p,q∈M such that $\log_{p}q=V\in T_{p}M$ is well defined. The differential at p of the mapping $m\mapsto d^{2}\left(m,q\right)$ is the 1-form $\alpha_{q}:X\in T_{p}M\mapsto 2g\left(X,V\right)$.*

**Proposition** **7** (Variational formula [37] p. 333, Theorem B.3). *Let X be a vector field depending on a parameter λ, with value at point p denoted as X(p;λ). Let $\Phi_{X}$ be its flow, i.e., $\Phi_{X}\left(t,x;\lambda \right)$ is the value of the integral curve of X at time t, with Φ(0,x;λ)=x. Then, the derivative $\partial_{\lambda }\Phi$ of *Φ* with respect to λ is the solution of the following:*

(34) $$\frac{d}{dt}\partial_{\lambda }\Phi_{X}\left(t,x;\lambda \right)=\partial_{x}X\left(\Phi (t,x;\lambda );\lambda \right)\partial_{\lambda }\Phi_{X}\left(t,x;\lambda \right)+\partial_{\lambda }X\left(\Phi_{X}\left(t,x;\lambda \right),\lambda \right)$$

*with the initial condition as follows:*

(35) $$\partial_{\lambda }\Phi_{X}\left(0,x;\lambda \right)=0.$$

*Similarly, the derivative with respect to the initial condition is the solution of the following:*

(36) $$\frac{d}{dt}\partial_{x}\Phi_{X}\left(t,x;\lambda \right)=\partial_{x}X\left(\Phi (t,x;\lambda );\lambda \right)\partial_{x}\Phi_{X}\left(t,x;\lambda \right)$$

*with the initial condition as follows:*

(37) $$\partial_{x}\Phi_{X}\left(0,x;\lambda \right)=\mathrm{Id}.$$

The variational equation can be applied to the geodesic equation $\nabla_{\dot{\gamma }}\dot{\gamma }=0$ by considering a perturbation in the derivative at 0 in the form $\lambda \mapsto \dot{\gamma }\left(0\right)+\lambda v$ while keeping the starting point p=γ(0) constant. The one-parameter family of curves will be denoted by γ(p,t;λ). Letting $J=\partial_{\lambda }\gamma$, we obtain the following:

(38) $$\frac{d^{2}}{dt^{2}}J^{i}=-\left(\partial_{l}\Gamma_{jk}^{i}\right){\dot{\gamma }}^{i}{\dot{\gamma }}^{j}J^{l}-2\Gamma_{jk}^{i}\frac{d}{dt}J^{j}{\dot{\gamma }}^{k},$$

where the 2 factor is a consequence of ∇ being torsionless. A more familiar form can be obtained by expanding *J* on a frame $X_{1}\left(t\right),\ldots X_{n}\left(t\right)$ coming from parallel transport of a basis of $T_{p}M$. In this case, letting $J\left(t\right)=J^{\alpha }\left(t\right)X_{\alpha }\left(t\right)$, Equation (38) becomes

(39) $$\frac{d^{2}}{dt^{2}}J+R^{\nabla }\left(J,\dot{\gamma }\right)\dot{\gamma }=0.$$

with $R^{\nabla }$ the curvature of ∇.

**Remark** **11.** *Equation (39) is valid for an arbitrary Koszul connection without torsion, even if it is a more common use in the Riemannian setting. A solution vector field J along γ is said to be a Jacobi vector field.*

**Remark** **12.** *The result is used locally before the injectivity radius of the exp function.*

Going back to the original problem, which is to find a local minimum of Criterion (32), and assuming that γ is the integral curve of a vector field *X* with the initial condition γ(0)=p, an application of Lemma 2 and Proposition 7 yields the next expression for the differential at $T_{p}M$ of E(γ) with respect to the initial conditions as follows:

(40) $$dE:Y\in T_{p}M\mapsto \left\{\begin{array}{cc} \; & \frac{2}{N}\sum_{i=1}^{N}g\left(\gamma \left(t_{i}\right);\partial_{p}\gamma \left(t_{i},p\right).Y,\log_{\gamma (t_{i},p)}y_{i}\right),\\ \; & \frac{2}{N}\sum_{i=1}^{N}g\left(\gamma \left(t_{i}\right);\partial_{v}\gamma \left(t_{i},p\right).Y,\log_{\gamma (t_{i},p)}y_{i}\right),\\ \; & v=\dot{\gamma }\left(0\right). \end{array}\right.$$

The Riemannian gradient at $T_{p}M$ is thus the tangent vector $Z_{0}\in T_{p}M$ (resp. $Z_{1}\in T_{p}M$) such that [27]

(41) $$\forall Y\in T_{p}M,\left\{\begin{array}{cc} \; & g\left(p;Y,Z_{0}\right)=\frac{2}{N}\sum_{i=1}^{N}g\left(\gamma \left(t_{i}\right);\partial_{p}\gamma \left(t_{i},p\right).Y,\log_{\gamma (t_{i},p)}y_{i}\right),\\ \; & g\left(p;Y,Z_{1}\right)=\frac{2}{N}\sum_{i=1}^{N}g\left(\gamma \left(t_{i}\right);\partial_{v}\gamma \left(t_{i},p\right).Y,\log_{\gamma (t_{i},p)}y_{i}\right). \end{array}\right.$$

Let, for i=1,…,N, the individual contributions of the gradient

(42) $$\begin{array}{cc} dE_{i}^{p} & :=g\left(\gamma \left(t_{i}\right);\partial_{p}\gamma \left(t_{i},p\right).Y,\log_{\gamma (t_{i},p)}y_{i}\right), \end{array}$$

(43) $$\begin{array}{cc} dE_{i}^{v} & :=g\left(\gamma \left(t_{i}\right);\partial_{v}\gamma \left(t_{i},p\right).Y,\log_{\gamma (t_{i},p)}y_{i}\right), \end{array}$$

**Figure 1:**
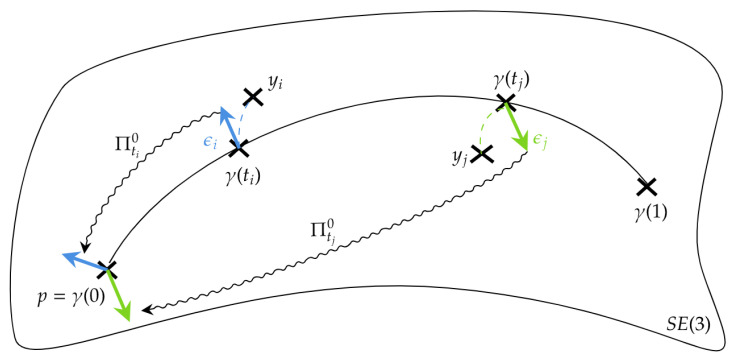
Parallel transport of the residues.

$\partial_{*}\gamma \left(t_{i},p\right).Y$ and $\log_{\gamma (t_{i},p)}y_{i}$ both belong to $T_{\gamma (t_{i})}M$. As illustrated in Figure 1 (where $\Pi_{t_{k}}^{0}$ denotes the parallel transport from $T_{\gamma (t_{k})}M$ to $T_{\gamma (0)}M$, and $\epsilon_{k}=\log_{\gamma (t_{k},p)}y_{k}$ denotes the residue at time $t_{k}$), we can transport them back to $T_{p}M$, so that there are two vectors $\left(Z_{0}^{i},Z_{1}^{i}\right)$ such that

(44) $$\begin{array}{cc} dE_{i}^{p} & =g\left(\gamma \left(t_{i}\right);\;\partial_{p}\gamma \left(t_{i},p\right).Y,\epsilon_{i}\right)=g\left(p;\;Y,Z_{0}^{i}\right), \end{array}$$

(45) $$\begin{array}{cc} dE_{i}^{v} & =g\left(\gamma \left(t_{i}\right);\;\partial_{v}\gamma \left(t_{i},p\right).Y,\epsilon_{i}\right)=g\left(p;\;Y,Z_{1}^{i}\right). \end{array}$$

The vector $Z_{0}$ (respectively, $Z_{1}$) can be evaluated by summing the individual contributions as follows:

(46) $$\forall Y\in T_{p}M,\left\{\begin{array}{cc} \; & g\left(p;\;Y,Z_{0}\right)=\frac{2}{N}\sum_{i=1}^{N}g\left(p;\;Y,Z_{0}^{i}\right)=\frac{2}{N}g\left(p;\;Y,\sum_{i=1}^{N}Z_{0}^{i}\right),\\ \; & g\left(p;\;Y,Z_{1}\right)=\frac{2}{N}\sum_{i=1}^{N}g\left(p;\;Y,Z_{1}^{i}\right)=\frac{2}{N}g\left(p;\;Y,\sum_{i=1}^{N}Z_{1}^{i}\right). \end{array}\right.$$

Finding each $Z_{0}^{i}$, (respectively, $Z_{1}^{i}$) boils down to inverting the matrix $\partial_{p}\gamma \left(t_{i},p\right)$ (respectively, $\partial_{v}\gamma \left(t_{i},p\right)$) and then transposing it with respect to the metric. However, an easier way to do this is by parallelly transporting the residue $\log_{\gamma (t_{i},p)}y_{i}$ from $T_{\gamma (t_{i})}M$ back to $T_{p}M$.

Following [24], the problem can be tackled with the introduction of the adjoint equations. While the original work was conducted with the Levi–Civita connection, the derivation of the adjoint equations can be performed mutantis mutandis using the dual-connection $\nabla^{*}$ as follows:  

(47) $$\begin{array}{cc} \; & \nabla_{\dot{\gamma }}^{*}\lambda_{i}\left(t\right)=-\lambda_{i-1}\left(t\right), \end{array}$$

(48) $$\begin{array}{cc} \; & \;\nabla_{\dot{\gamma }}^{*}\lambda_{0}\left(t\right)=-\sum_{i}R^{\nabla^{*}}\left(v_{i}\left(t\right),\lambda_{i}\left(t\right)\right)v_{1}\left(t\right), \end{array}$$

where the $v_{i}$ values are the vector fields solutions of Equation (30), and the $\lambda_{i}$ values are adjoint vector fields coupled with the $v_{i}$ values.

### 5.3. Application to SE(3)

Coming back to the original problem, which is to estimate a trajectory (i.e., a time-stamped curve) from raw positioning data belonging to SE(3), we remind here some specific elements about SE(n) and its Lie algebra se(n).

**Definition** **19.** *The set of affine maps $f:R^{n}\to R^{n}$ such that f(x)=Rx+T where R is a rotation matrix (R∈SO(n)), and T is a vector of $R^{n}$ is called the Special Euclidean group SE(n) and is a Lie group.*

SE(n) is often called the group of rigid motions, or rigid-body motions. An element A∈SE(n) can be represented by a (n + 1)-by-(n + 1) matrix of the form $\left(\begin{array}{cc} R & T\\ 0 & 1 \end{array}\right)$ where R∈SO(n) and $T\in R^{n}$ It is a group, where the product of two elements of SE(3) is given as follows:

$$\left(\begin{array}{cc} R_{1} & T_{1}\\ 0 & 1 \end{array}\right)\times \left(\begin{array}{cc} R_{2} & T_{2}\\ 0 & 1 \end{array}\right)=\left(\begin{array}{cc} R_{1}R_{2} & R_{1}T_{2}+T_{1}\\ 0 & 1 \end{array}\right)$$

and the inverse of an element is as follows:

$${\left(\begin{array}{cc} R & T\\ 0 & 1 \end{array}\right)}^{-1}=\left(\begin{array}{cc} R^{T} & -R^{T}T\\ 0 & 1 \end{array}\right)$$

We have $\dim\left(SE\left(n\right)\right)=\frac{n(n+1)}{2}$; therefore, dim(SE(3))=6.

**Definition** **20.** *The vector space of real (n + 1)-by-(n + 1) matrices of the form*

(49) $$X=\left(\begin{array}{cc} K & V\\ 0 & 0 \end{array}\right)$$

*where K is a skew-symmetric n-by-n matrix and V is a vector in $R^{n}$ and the Lie algebra of SE(n).*

**Remark** **13.** *It can be easily shown that the matrix exponential of any matrix defined by (49) belongs to SE(n). It can also be proven by the exp map se(n)→SE(n) is onto but not one-to-one due to the rotation part of the matrix being the same with angles differing by an integer multiple of 2π.*

Since dim(se(3))=dim(SE(3))=6, a basis for se(3) corresponding to the infinitesimal rotations and translations along all three axes can be written as follows:

$$\begin{array}{c} E_{1}=\left(\begin{array}{cccc} 0 & 0 & 0 & 0\\ 0 & 0 & -1 & 0\\ 0 & 1 & 0 & 0\\ 0 & 0 & 0 & 0 \end{array}\right),E_{2}=\left(\begin{array}{cccc} 0 & 0 & 1 & 0\\ 0 & 0 & 0 & 0\\ -1 & 0 & 0 & 0\\ 0 & 0 & 0 & 0 \end{array}\right),E_{3}=\left(\begin{array}{cccc} 0 & -1 & 0 & 0\\ 1 & 0 & 0 & 0\\ 0 & 0 & 0 & 0\\ 0 & 0 & 0 & 0 \end{array}\right),\\ E_{4}=\left(\begin{array}{cccc} 0 & 0 & 0 & 1\\ 0 & 0 & 0 & 0\\ 0 & 0 & 0 & 0\\ 0 & 0 & 0 & 0 \end{array}\right),E_{5}=\left(\begin{array}{cccc} 0 & 0 & 0 & 0\\ 0 & 0 & 0 & 1\\ 0 & 0 & 0 & 0\\ 0 & 0 & 0 & 0 \end{array}\right)E_{6}=\left(\begin{array}{cccc} 0 & 0 & 0 & 0\\ 0 & 0 & 0 & 0\\ 0 & 0 & 0 & 1\\ 0 & 0 & 0 & 0 \end{array}\right) \end{array}$$

There is a canonical isomorphism between se(3) and $R^{6}$ often denoted ∧ (“hat”), with its inverse being denoted ∨ (“vee”) as follows:

$$\wedge :R^{6}\to \mathrm{se}\left(3\right),\vee :\mathrm{se}\left(3\right)\to R^{6}$$

With these elements in mind, we can solve the regression. Ref. [24] gave a closed-form expression for geodesics in groups like SO(3) that have a bi-invariant metric, but no such closed form can be derived for a higher-order polynomial. Unfortunately, SE(3) has no such bi-invariant metric [11], and numerical integration is the only solution available, even for order 1 polynomials, i.e., geodesics.

## 6. Implementation

### Algorithms

We now show how the solution is numerically implemented.

The algorithm was tested on several generic trajectories generated with the MatLab software, with different noise variances according to the pseudo-code presented in Algorithm 2. In this algorithm, a maneuver is defined as a change in the motion or orientation of the object, altering its speed and direction with respect to a fixed reference point. A maneuver can be as simple as a turn, a climb, a descent, or something more complex such as a loop or helix.

Note that, here, for a given maneuver, the velocity in se(3) is assumed to be constant during the said maneuver; otherwise, we would have to integrate the velocity vector field over time, as in Equation (26).

The metric used is the one derived from the usual inner product in matrix spaces:

$$\langle A,B\rangle =tr\left(A^{T}B\right)$$

We used the Levi–Civita connection for its compatibility with the metric to measure the differences between the actual, noisy, and estimated trajectories. The algorithm may easily be changed to account for another connection.

The pseudocode in Algorithm 3 describes the procedure for determining the optimal geodesic but can easily be extended to determine an optimal curve of order *k*.

The following factors should be noted:

- Initialization of p=γ(0) may be carried out in several ways: naive initialization $p_{0}=e$ may work, but the Fréchet mean ([38]) is often a better alternative. Another possibility is to use one of the measured points;
- Equations (33) are solved in Algorithm 3 in the specific case of a geodesic, which means k=1 as follows: we may rewrite the forward equations using the Christoffel symbols of the connection and then solve a “large” system of ODEs.
  $$\begin{array}{ccc} \left\{\begin{array}{ccc} \frac{d}{dt}\gamma \left(t\right) & = & \gamma \left(t\right)X_{1}\left(t\right)\\ \; & \vdots & \\ \frac{d}{dt}X_{i}\left(t\right) & = & {\bar{\nabla }}_{X_{1}}X_{i}\left(t\right)+X_{i+1}\left(t\right)\\ \; & \vdots & \\ \frac{d}{dt}X_{k}\left(t\right) & = & {\bar{\nabla }}_{X_{1}}X_{k}\left(t\right) \end{array}\right.\; & \Rightarrow & \left\{\begin{array}{ccc} \gamma (t) & = & \exp\left(\int_{0}^{t}X_{1}\left(u\right)du\right)\\ \; & \vdots & \\ {\dot{X}}_{i}\left(t\right) & = & \Gamma_{lj}^{m}X_{1}^{l}X_{i}^{j}+X_{i+1}\\ \; & \vdots & \\ {\dot{X}}_{k}\left(t\right) & = & \Gamma_{lj}^{m}X_{1}^{l}X_{k}^{j} \end{array}\right. \end{array}$$

**Algorithm 2:** Simulated data generation
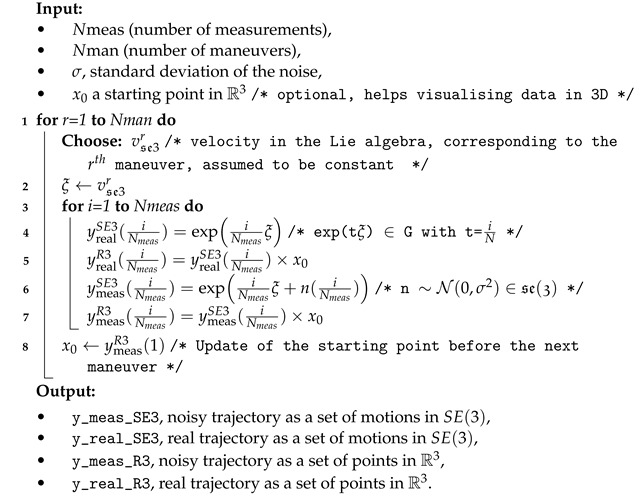


**Algorithm 3:** Determining the optimum geodesic

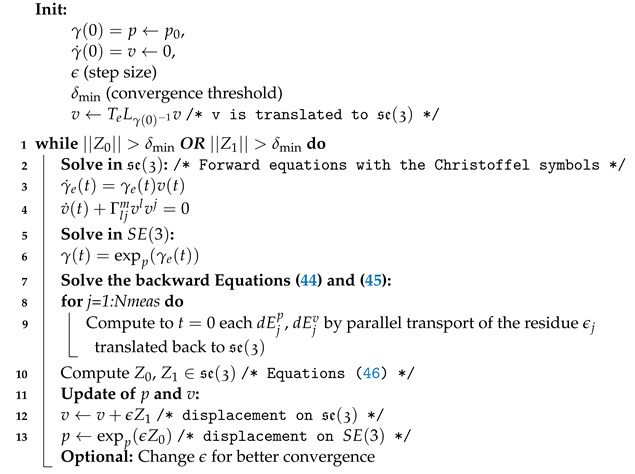



**Figure 2:**
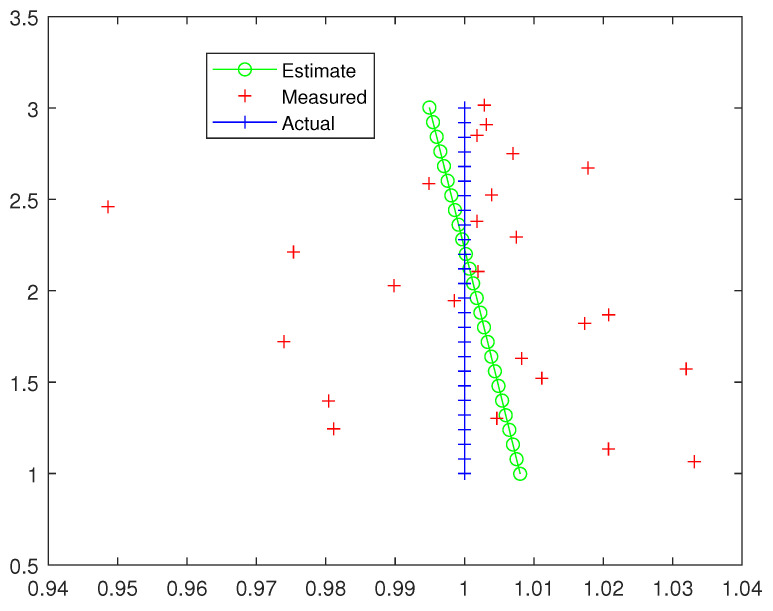
A straight line.

**Figure 3:**
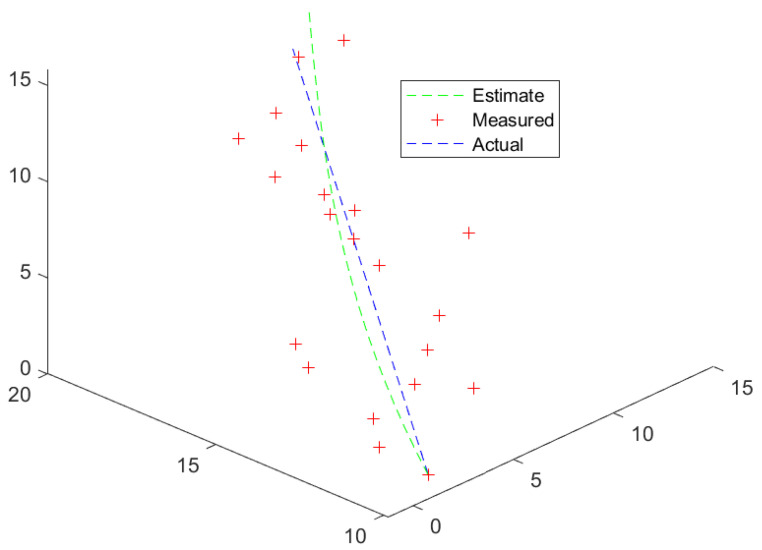
A straight line, with a “large” additive noise.

The first examples are shown in Figure 2 and Figure 3. We can see that the estimate is close to the actual trajectory, even though a slightly curving move is present due to the estimated rotation part of the trajectory in SE(3) being close to zero but not exactly zero. This could be corrected by post-processing by the flagging of the trajectory.

**Figure 4:**
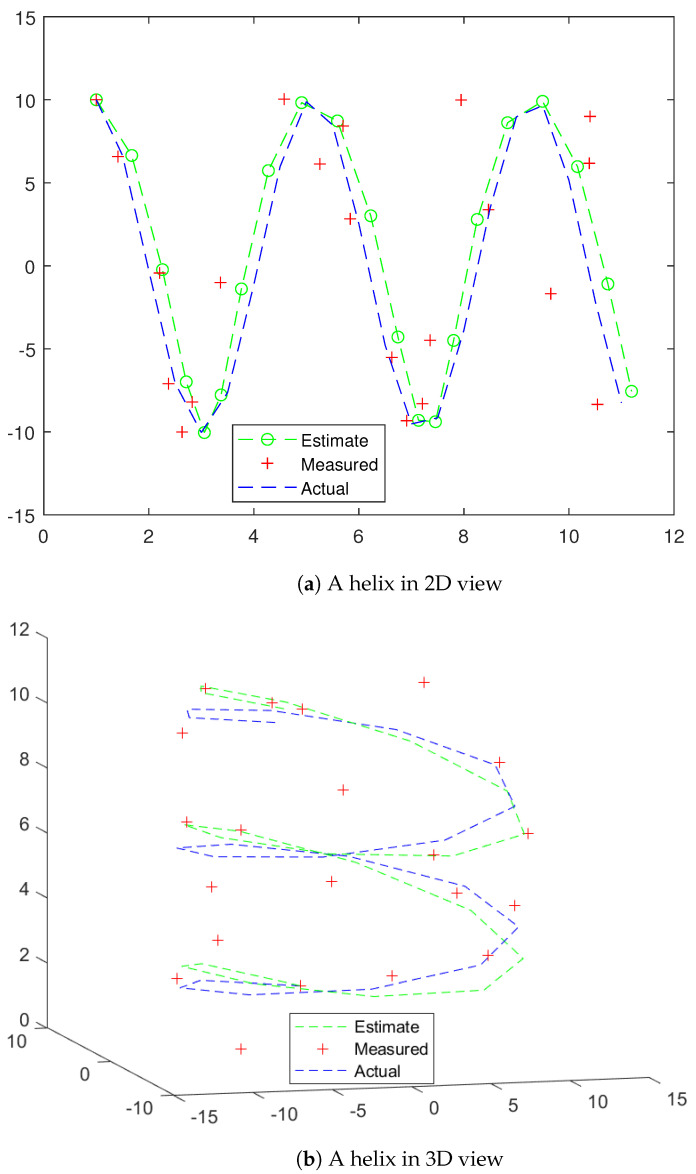
A helix.

**Figure 5:**
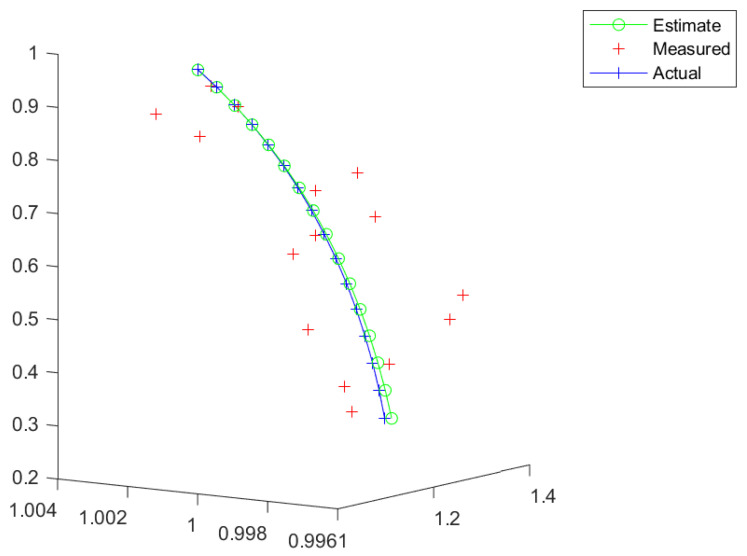
A gradual turn.

**Figure 6:**
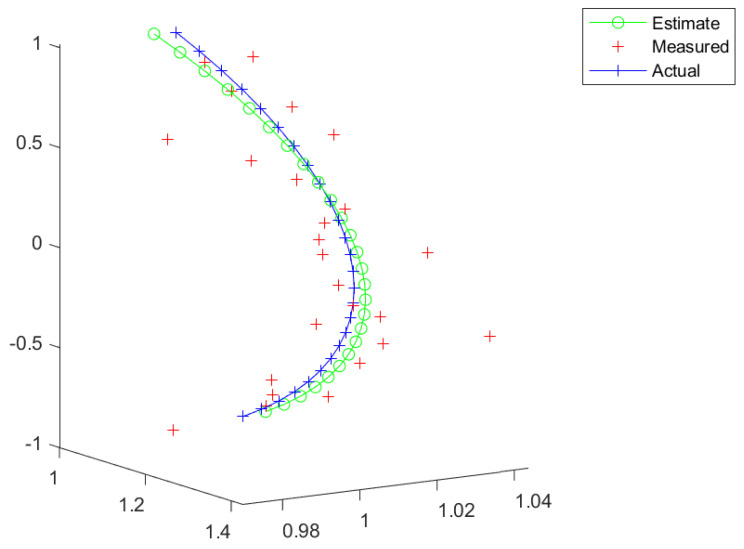
A sharp turn.

Figure 4 displays a trajectory that is highly unlikely for a civilian aircraft but could be plausible for a UAV. This kind of trajectory would be difficult to track by a Kalman filter unless the state variables also lie on a Lie group. Also, turn maneuvers were tested as shown in Figure 5 and Figure 6.

We can measure how well the model fits the data using a coefficient of determination $R^{2}$. Following [27], since the data do not belong to an Euclidean space, we will have to use the Fréchet variance as the denominator as follows:

$${\mathrm{Var}}_{F}=\frac{1}{N}\min_{y\in M}\sum_{j}d{\left(y,y_{j}\right)}^{2},$$

leading to

(50) $$R^{2}=1-\frac{SSE}{\mathrm{sample}\;\mathrm{Fr}é\mathrm{chet}\;\mathrm{variance}}=1-\frac{\sum_{j}d{\left(\gamma \left(t_{j}\right),y_{j}\right)}^{2}}{\min_{y\in M}\sum_{j}d{\left(y,y_{j}\right)}^{2}}$$

Note that since we use simulated data, we can also measure the $R^{2}$ coefficient with respect to the “real” data:

(51) $$R^{2}=1-\frac{\sum_{j}d{\left(\gamma \left(t_{j}\right),y_{j}^{\mathrm{real}}\right)}^{2}}{\min_{y\in M}\sum_{j}d{\left(y,y_{j}^{\mathrm{real}}\right)}^{2}}$$

Similarly to the Euclidean case, an $R^{2}$ close to 0 will indicate that the estimator does not provide a better estimate than the sample mean (Fréchet mean for manifold-valued data), whereas an $R^{2}$ equal to 1 will indicate a perfect fit between the model and the data.

Some of the results obtained from the simulations are provided in Table 1.

We notice that the algorithm performs rather well even with “sharp” maneuvers, even though the performance degrades when the noise variance increases, which makes total sense. The algorithm seems to perform well even with a small sample size such as 10 points. The nature of the maneuver does not seem to affect the performance. The source code and more data sets are available as Supplementary Material.

## 7. Conclusions and Future Work

In this work, we have written polynomial regression in Lie groups as a solution of a *k*-times-iterated covariant derivative. For that, the iterated connection is not necessarily the Levi–Civita connection. The solution was tested in the specific case of the Special Euclidean group SE(3) that models rigid-body motions.

From that, several possibilities may be pursued. A theoretical study of the performance of the estimator in terms of convergence could be undertaken. Also, since the connection exponential defines a local coordinate system, we can go further and find coordinate systems that flatten the manifold, thus greatly simplifying the regression problem and having it behave as in the Euclidean case. Further work would consist of characterizing the statistical quantities associated with the estimation process using polynomial regression. Determining the limit law to be considered in this case is a problem in itself. Another axis would involve using local *G*-valued polynomials to track the changes in maneuvers and adapt the parameters of the optimum fitting curve.   

## Figures and Tables

**Table 1:** Results after simulations.

Maneuver	Noise Std	Nmeas	$R^{2}$
Straight line	$10^{-3}$	25	1
**Straight line**	$10^{-2}$	**25**	**0.99**
Straight line	$10^{-1}$	25	0.84
Straight line	$10^{-2}$	25	0.99
Straight line	$10^{-2}$	15	0.99
Straight line	$10^{-2}$	10	0.99
Straight line	$10^{-1}$	25	0.84
Straight line	$10^{-1}$	15	0.82
Straight line	$10^{-1}$	10	0.81
Gradual turn	$10^{-2}$	25	0.99
**Gradual turn**	$10^{-2}$	**15**	**0.99**
Gradual turn	$10^{-2}$	10	0.99
Gradual turn	$10^{-1}$	25	0.79
Gradual turn	$10^{-1}$	15	0.75
Gradual turn	$10^{-1}$	10	0.74
**Sharp turn**	$10^{-1}$	**25**	**0.92**
Sharp turn	$10^{-1}$	15	0.89
Sharp turn	$10^{-1}$	10	0.81

Note: the bold lines are illustrated by images.

## Data Availability

The original data presented in the study are openly available in GitHub at https://github.com/jhnaby/LieGroupRegression (accessed on 26 September 2024).

## Supplementary Materials

All MATLAB scripts and some datasets are freely available at https://github.com/jhnaby/LieGroupRegression (accessed on 26 September 2024).

## References

[1] Julier S.J., Uhlmann J.K., Kadar I. (1997). New extension of the Kalman filter to nonlinear systems. Proceedings of the Signal Processing, Sensor Fusion, and Target Recognition VI.

[2] Bourmaud G., Mégret R., Giremus A., Berthoumieu Y. Discrete Extended Kalman Filter on Lie groups. Proceedings of the 21st European Signal Processing Conference (EUSIPCO 2013).

[3] Phogat K.S., Chang D.E. (2020). Invariant extended Kalman filter on matrix Lie groups. Automatica.

[4] Bonnabel S. Left-invariant Extended Kalman Filter and attitude estimation. Proceedings of the 2007 46th IEEE Conference on Decision and Control.

[5] Bonnabel S., Martin P., Salaün E. Invariant Extended Kalman Filter: Theory and application to a velocity-aided attitude estimation problem. Proceedings of the 48h IEEE Conference on Decision and Control (CDC) Held Jointly with 2009 28th Chinese Control Conference.

[6] Fang K., Cai T., Wang B. (2024). The Kinematic Models of the SINS and Its Errors on the SE(3) Group in the Earth-Centered Inertial Coordinate System. Sensors.

[7] Jeong D.B., Lee B., Ko N.Y. (2024). Three-Dimensional Dead-Reckoning Based on Lie Theory for Overcoming Approximation Errors. Appl. Sci..

[8] Sun J., Chen Y., Cui B. (2024). An Improved Initial Alignment Method Based on SE2(3)/EKF for SINS/GNSS Integrated Navigation System with Large Misalignment Angles. Sensors.

[9] Park F., Bobrow J., Ploen S. (1995). A Lie Group Formulation of Robot Dynamics. Int. J. Robot. Res..

[10] Wang W., Peng X., Ai J., Fu C., Li C., Zhang Z. (2024). SE(3) Based LTV-MPC Algorithm for Multi-Obstacle Trajectory Tracking of Fully Driven Spacecraft. IEEE Access.

[11] Miolane N., Pennec X. (2015). Computing Bi-Invariant Pseudo-Metrics on Lie Groups for Consistent Statistics. Entropy.

[12] Boisvert J., Pennec X., Ayache N., Labelle H., Cheriet K. 3D anatomical variability assessment of the scoliotic spine using statistics on Lie groups. Proceedings of the 3rd IEEE International Symposium on Biomedical Imaging: Nano to Macro.

[13] Boisvert J., Cheriet F., Pennec X., Labelle H., Ayache N. (2008). Geometric Variability of the Scoliotic Spine Using Statistics on Articulated Shape Models. IEEE Trans. Med Imaging.

[14] Hanik M., Hege H.C., von Tycowicz C. (2022). Bi-Invariant Dissimilarity Measures for Sample Distributions in Lie Groups. Siam J. Math. Data Sci..

[15] Fiori S., Rossi L.D. (2022). Minimal control effort and time Lie-group synchronisation design based on proportional-derivative control. Int. J. Control.

[16] Duan X., Sun H., Zhao X. (2019). A Matrix Information-Geometric Method for Change-Point Detection of Rigid Body Motion. Entropy.

[17] Fiori S. (2022). Manifold Calculus in System Theory and Control—Second Order Structures and Systems. Symmetry.

[18] Smith S. (2005). Covariance, subspace, and intrinsic Cramer-Rao bounds. IEEE Trans. Signal Process..

[19] Labsir S., Renaux A., Vilà-Valls J., Chaumette É. Cramér-Rao Bound on Lie Groups with Observations on Lie Groups: Application to SE(2). Proceedings of the ICASSP 2023—2023 IEEE International Conference on Acoustics, Speech and Signal Processing (ICASSP).

[20] Labsir S., Giremus A., Yver B., Benoudiba–Campanini T. (2024). An intrinsic Bayesian bound for estimators on the Lie groups SO(3) and SE(3). Signal Process..

[21] Jeon J.M., Park B.U., Keilegom I.V. (2022). Nonparametric regression on Lie groups with measurement errors. Ann. Stat..

[22] Camarinha M., Silva Leite F., Crouch P. (2001). On the geometry of Riemannian cubic polynomials. Differ. Geom. Its Appl..

[23] Camarinha M., Silva Leite F., Crouch P. (2023). High-Order Splines on Riemannian Manifolds. Proc. Steklov Inst. Math..

[24] Hinkle J., Muralidharan P., Fletcher P.T., Joshi S., Fitzgibbon A., Lazebnik S., Perona P., Sato Y., Schmid C. (2012). Polynomial Regression on Riemannian Manifolds. Proceedings of the Computer Vision—ECCV 2012.

[25] Popiel T., Noakes L. (2007). Bézier curves and C2 interpolation in Riemannian manifolds. J. Approx. Theory.

[26] Hanik M., Hege H.C., Hennemuth A., von Tycowicz C., Martel A.L., Abolmaesumi P., Stoyanov D., Mateus D., Zuluaga M.A., Zhou S.K., Racoceanu D., Joskowicz L. (2020). Nonlinear Regression on Manifolds for Shape Analysis using Intrinsic Bézier Splines. Proceedings of the Medical Image Computing and Computer Assisted Intervention—MICCAI 2020.

[27] Fletcher P.T., Pennec X., Joshi S., Nielsen M. Geodesic Regression on Riemannian Manifolds. Proceedings of the Third International Workshop on Mathematical Foundations of Computational Anatomy—Geometrical and Statistical Methods for Modelling Biological Shape Variability.

[28] Pennec X., Nielsen F., Barbaresco F. (2013). Bi-invariant Means on Lie Groups with Cartan-Schouten Connections. Proceedings of the Geometric Science of Information.

[29] Amari S.I., Nagaoka H. (2000). Methods of Information Geometry.

[30] Husemöller D. (2013). Fibre Bundles.

[31] Willmore T. (1996). Riemannian Geometry.

[32] Agrachev A.A., Sachkov Y.L. (2004). Control Theory from the Geometric Viewpoint.

[33] Saunders D.J. (1989). The Geometry of Jet Bundles.

[34] Boumal N. (2023). An Introduction to Optimization on Smooth Manifolds.

[35] Marsden J., Ratiu T. (1999). Introduction to Mechanics and Symmetry: A Basic Exposition of Classical Mechanical Systems.

[36] Niethammer M., Huang Y., Vialard F.X. Geodesic Regression on Image Time Series. Proceedings of the Medical Image Computing and Computer-Assisted Intervention: MICCAI—International Conference on Medical Image Computing and Computer-Assisted Intervention.

[37] Duistermaat J., Kolk J. (1999). Lie Groups.

[38] Fréchet M. (1948). Les éléments aléatoires de nature quelconque dans un espace distancié. Ann. L’Institut Henri PoincarÉ.

